# Multimodality Imaging of Pelvic Inflammatory Disease Complicated with Tubo-Ovarian Abscess

**DOI:** 10.5334/jbsr.3061

**Published:** 2023-03-28

**Authors:** Guillaume Puissant, Latifa Fellah, Vasiliki Perlepe

**Affiliations:** 1 Cliniques universitaires Saint-Luc, BE

**Keywords:** Pelvic Inflammatory disease, Multimodality imaging, Pelvis

## Abstract

**Teaching Point:** Pelvic inflammatory disease (PID) is the most frequent gynecologic cause of emergency visits. Because of its prevalence and non-specific symptoms, the radiologist may encounter this pathology and its complications on all imaging modalities and should carefully assess PID signs to avoid delay in management, late complications, and unnecessary surgery.

## Case History

**Figure 1:**
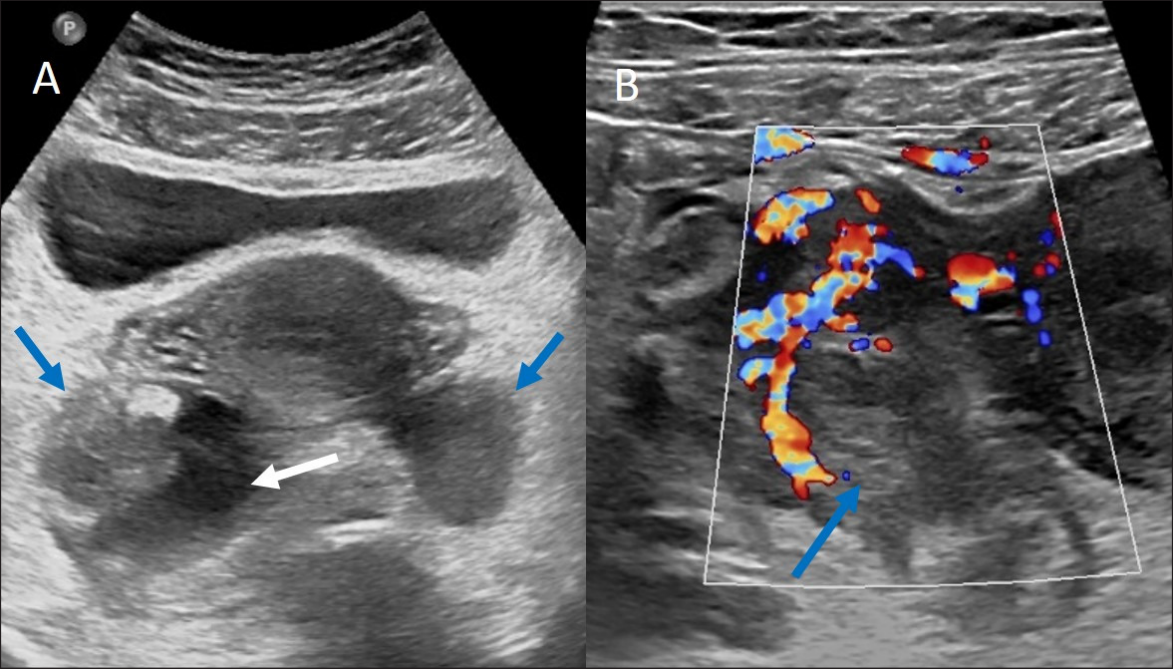


A 32-year-old female patient hospitalized for treatment of a B-cell lymphoma presented fever for a week and abdominal pain for two days with pelvic and right iliac fossa tenderness. Blood tests revealed signs of infection (leukocytosis and elevated CRP). The patient first underwent abdominal ultrasound (Figure 1) showing bilateral adnexal swelling (Figure 1A, blue arrows) and a right ovarian heterogeneous partially cystic mass (Figure 1A, white arrow). It also showed a thick-walled hyperemic right fallopian tube (Figure 1B, blue arrow).

**Figure 2:**
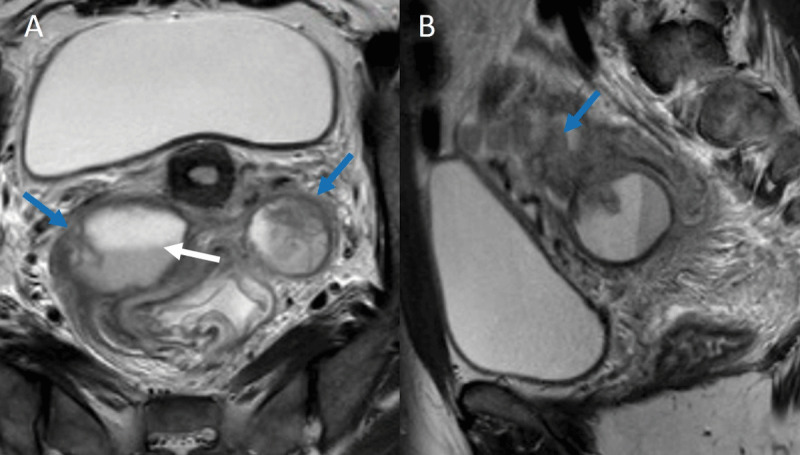


Magnetic resonance imaging (MRI) confirmed the diagnosis of tubo-ovarian abscess. Axial and sagittal T2 weighted images (Figure 2) showed bilateral complex cystic adnexal mass (Figure 2A, blue arrows) with peri-ovarian fat stranding and right fluid-fluid level corresponding to intra-ovarian declive pus (Figure 2A, white arrow). It also revealed dilated fallopian tube filled with complex internal fluid (Figure 2B, blue arrow) corresponding to pyosalpinx.

**Figure 3:**
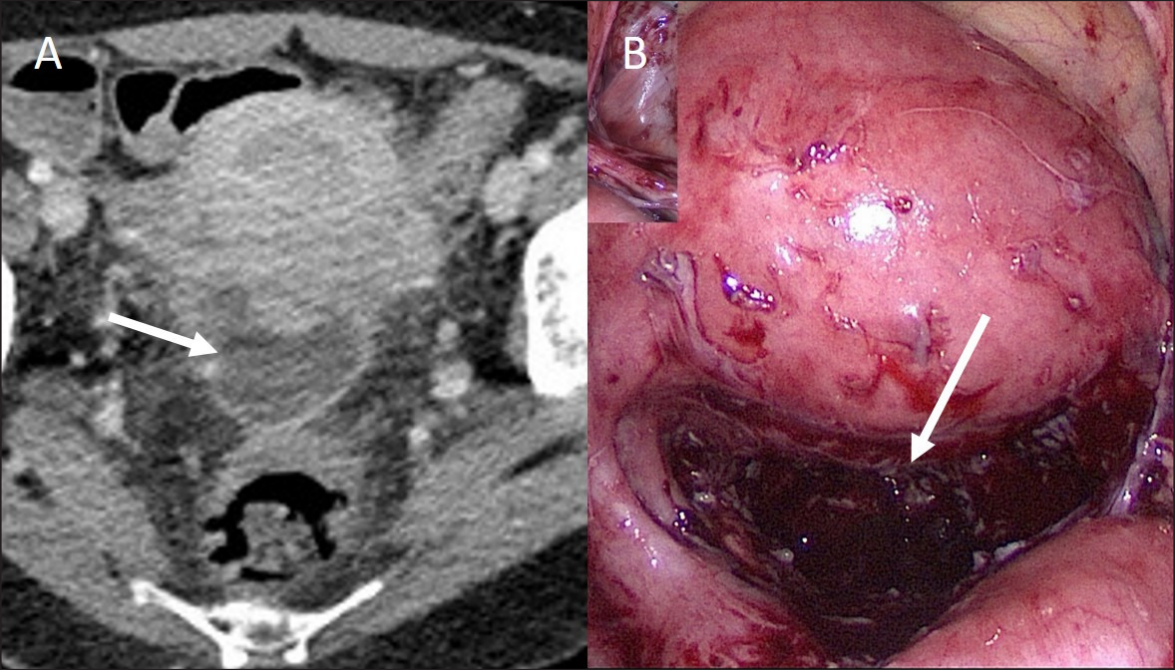


A week later, the patient presented to the emergency room while under antibiotherapy for persistent pelvic pain. A computed tomography (CT) scan was performed and revealed a persistent tubo-ovarian abscess with extension to the recto-uterine pouch (Figure 3A, white arrow). Laparoscopy confirmed the diagnosis (Figure 3B, white arrow).

## Comments

Pelvic inflammatory disease is a common infection in young women, affecting 4.4% of women between 18 to 44 years old. CT is often the first imaging modality for the evaluation of these patients in the emergency department because the presenting symptoms are vague and non-specific. TDM early signs of PID include thickening of the uretero-sacral ligament, free fluid in the cul-de-sac, pelvic fat infiltration, and reactive adenopathy. Thickening of the fallopian tubes (>5 mm) was found to have specificity of 95% for PID. Early detection of PID can reduce the risk of tube-related infertility, ectopic pregnancy, and pelvic chronic pain due to adhesions [1].

Ultrasonography is considered the first-line imaging modality in the evaluation of suspected salpingitis showing free fluid in the cul-de-sac and thickening and wall enhancement of the fallopian tubes [1].

In advanced cases of PID, MRI is more sensitive for the detection of ovarian involvement, differentiation between pyosalpinx and hematosalpinx, and the differential diagnosis with ovarian malignancy [1].

## References

[1] Revzin, MV, Mathur, M, Dave, HB, Macer, ML, Spektor, M. Pelvic inflammatory disease: Multimodality imaging approach with clinical-pathologic correlation. RadioGraphics. 2016; 36(5): 1579–1596. DOI: 10.1148/rg.2016150202 27618331

